# Gender Differences in the Social Pathways Linking Neighborhood Disadvantage to Depressive Symptoms in Adults

**DOI:** 10.1371/journal.pone.0076554

**Published:** 2013-10-17

**Authors:** Emma Bassett, Spencer Moore

**Affiliations:** 1 School of Kinesiology and Health Studies, Queen’s University, Kingston, Ontario, Canada; 2 Department of Community Health and Epidemiology, Queen’s University, Kingston, Ontario, Canada; Chancellor College, University of Malawi, Malawi

## Abstract

Depression debilitates the lives of millions and is projected to be the second leading disease burden worldwide by 2020. At the population level, the causes of depression are found in the everyday social and physical environments in which people live. Research has shown that men and women often experience neighbourhood environments differently and that these variations are often reflected in health outcomes. The current study examines whether social and environmental correlates of depression are similar in men and women. This study examines whether (i) there are gender differences in the association between neighbourhood disadvantage and depressive symptoms, and (ii) dimensions of social capital and cohesion mediate these associations. Data come from the Montreal Neighbourhood Networks and Healthy Aging Study, which consists of a cluster stratified sample of Montreal census tracts (n_ct_ = 300) and individuals within those tracts (n_i_ = 2707). Depressive symptoms and social capital were measured with a questionnaire. Neighbourhood disadvantage was measured at the census tract level using data from the 2006 Canada Census. Multilevel logistic regression stratified by gender and a three-step mediation analysis procedure were used. Final sample size for these analyses was 2574 adults. Depressive symptoms had a prevalence of 17.3% in the overall sample. Disadvantage was associated with depressive symptoms in women only (OR = 1.25, 95% CI = 1.01–1.55). Perceived neighbourhood cohesion was shown to mediate the association of disadvantage and depressive symptoms in women (ab = 0.02; 95% CI = 0.003–0.04, p<0.05). Other socio-relational variables, specifically generalized trust and trust in neighbours were associated with depression in women but did not act as mediating variables. Health promotion initiatives meant to combat depression may wish to consider gender differences in the design and implementation of neighbourhood or peer-based programs.

## Introduction

Depression is a major health concern in countries around the world, and has a significant impact on the lives of millions of Canadians [1]. Major depressive disorder includes symptoms such as depressed mood, loss of pleasure, and loss of interest in nearly all daily activities [2]. According to the Canadian Community Health Survey, Cycle 1.2, 10.8% of Canada’s population had experienced major depression in their lifetime [1]. Canadian prevalence rates of major depressive episodes in 2002 were 5% in women and 2.9% in men [1]. Research on gender and depression has consistently found that the rates of depression in women are typically twice those of men [3].

Differences in rates of depression between men and women may largely be attributed to social, psychological, and environmental factors [3]–[6]. Women, for example, have been identified as potentially more vulnerable than men to certain aspects of the neighbourhood environment [7]. Explanations of why neighbourhood environments may have different health effects on women and men include: (1) women and men perceive neighbourhoods differently; (2) women and men are exposed within neighbourhoods to different stressors and at varying degrees; and (3) women may be more vulnerable than men to certain aspects of the neighbourhood environment due to differences in social roles [7]. Despite such propositions, little research has examined whether characteristics of neighbourhood environments are more strongly associated with depressive symptoms in women than men. By examining potential differences in the association among neighbourhood disadvantage, social capital, and depressive symptoms in men and women, the following study seeks to contribute to our knowledge on the importance of neighbourhood conditions to the mental health and well being of adults. Given the generally higher rates of depressive symptoms in women, such knowledge might inform the design of neighbourhood-based interventions aiming to improve the mental health of women.

### Neighbourhood Disadvantage and Depression

Neighbourhood disadvantage refers to residential environments characterized by poverty, lack of access to employment, and reduced economic prospects [8]. Disadvantage measures have typically included information of local area characteristics such as percentage of single-mother households, unemployed residents, immigrants, or renters within neighbourhoods. Such measures are postulated to represent factors related to local, socioeconomic hardship and marginalization [9]–[12] For example, a high percentage of renters may reflect low rates of home ownership – a factor that is closely related to wealth, increased social accountability, and increased integration into the neighbourhood [12]. Studies on the relationship between neighbourhood disadvantage and depression have reported mixed results. Several studies have shown neighbourhood disadvantage to be associated with depressive symptoms [10], [13]–[16]. For example, a national U.S. study reported that neighbourhood disadvantage was associated with diagnosed major depressive disorder [10]. Other research has not found such associations after adjustment for other individual- or neighbourhood-level factors [17], [18]. Mixed findings may be due to a number of factors, including different macro-political contexts, measures of disadvantage, and study populations.

### Neighbourhood Social Cohesion or Social Capital as a Mediating Factor?

Neighbourhood disadvantage is seen as a characteristic of places and not individuals. As such, one of the key questions in research on neighbourhood disadvantage and health concerns the mechanisms by which disadvantage “gets under the skin.” Carpiano (2006) suggests that neighbourhood structural conditions impact health through their impact on neighbourhood social cohesion and social capital [19]. Neighbourhood social cohesion refers to patterns of social interaction and values (e.g., familiarity, trust) within neighbourhood settings, whereas neighbourhood social capital refers to the resources that are embedded within neighbourhood social networks [19]. Research on the importance of personal social capital for reducing the risk of depression has shown both cohesion and capital aspects associated with depressive symptoms. In terms of perceived social cohesion, studies have found generalized trust, trust in neighbours, social support, and neighbourhood social cohesion to be inversely related to depressive symptoms [13], [15], [20]–[24].

In terms of social networks and social capital, individuals with no close ties have been shown to report higher rates of depression than those who report a greater number of close ties [20]–[24]. Studies have also suggested that depression spreads within social networks with individuals who are connected to others with depression at an increased risk of developing depressive symptoms themselves [25]. Research on localized social relationships and depressive symptoms have indicated that perceived social cohesion, e.g., knowing one’s neighbours, is associated with fewer depressive symptoms [15], [26]–[27]. Yet, little is known about the potential role that actual neighbourhood or non-neighbourhood core ties in mediating the association of neighbourhood environmental characteristics on depressive symptoms.

Preliminary findings in this area suggest that social capital may mediate the association between neighbourhood characteristics and depressive symptoms [16], [26], [28]. For example, Haines et al. showed that network capital mediated the association between neighbourhood disadvantage and depressive symptoms among adults in the southern United States [9]. In addition, Kruger et al. reported that individual perceptions of the neighbourhood (i.e., perceived neighbourhood social capital, crime, and satisfaction) and social contact with neighbours mediated the relationship between neighbourhood deterioration and depressive symptoms [28]. Such findings highlight the importance of examining the social pathways linking disadvantage to depressive symptoms in men and women.

Due to the potential confounding of associations among neighbourhood disadvantage, social capital and depressive symptoms by gender, this study examined the associations between neighbourhood disadvantage and depressive symptoms in men and women separately. Three research questions guide the study: (1) Is neighbourhood disadvantage associated with depressive symptoms in men and women?; (2) does the association of neighbourhood social cohesion and social capital with depressive symptoms differ in women and men?; and (3) do specific dimensions of social cohesion or network capital mediate the association between neighbourhood disadvantage and depression in men and women?

## Methods

### Ethics Statement

Prior to taking part in the study’s telephone interview, participants were read the study’s letter of information and consent form. If individuals gave verbal consent to participate, they were administered the questionnaire by trained interviewers. Verbal consent was recorded on the computer-administered telephone interviewing system. This interview protocol was documented in the study’s ethics application. Ethics approval for the study was given by the Committee of Scientific Evaluation and Research Ethics of the Centre de Recherche at the Centre Hospitalier de l’Université de Montréal (CHUM) in October 2007 (N.D. 07.049).

### Study Design

Data come from the Montreal Neighbourhood Networks and Healthy Aging Study (MoNNET-HA). MoNNET-HA used a random stratified cluster sampling design to survey 2707 adults residing in 300 different Montreal Metropolitan Area (MMA) census tracts. Montreal census tracts (N = 862) were stratified into tertiles of low, medium, and high SES areas based on median household income data from the 2006 Canada Census. One hundred census tracts were randomly selected from each tertile for a total of 300 tracts. Within each tract, household selection was stratified by the age category of the household respondent so that three adults were selected from the following categories: (1) 25–44 years old; (2) 45–64; and (3) 65 and older. One participant per household was selected. Inclusion criteria for respondents specified that respondents were not currently institutionalized, that they were able to complete questionnaires in French or English, and that they had lived in their current residence for at least one year [29].

### Measures

This study uses individual and neighbourhood-level data. The MoNNET-HA study provides the individual level information on depressive symptoms, social capital, and the socio-demographic characteristics of study participants. Neighbourhood-level information is from the 2006 Canada Census.

#### Depressive symptoms

The Center for Epidemiologic Studies 10-item Depression Scale (CES-D-10 Scale) was used to measure depressive symptoms in our sample. The CES-D-10 scale asked respondents whether or not they had experienced depressive symptoms in the past week with each item eliciting a “yes” or “no” response [30]. For example, items included statements such as “I felt sad” or “I enjoyed life” with respondents asked to report whether they had or had not experienced the particular symptom during the past two weeks. These items were randomly ordered on the scale except for the final question, which was “I felt depressed.” Following validated diagnostic criteria, participants who responded affirmatively to four or more items were classified as having depressive symptoms [30]. The CES-D-10 scale has scored high on both reliability and validity and has been shown suitable for use in general adult populations [30]. The MoNNET-HA depression scale had a Cronbach’s alpha of 0.72.

#### Network social capital

Core tie diversity was used to represent network social capital. A name generator asking respondents to list up to three alters with whom they had discussed important matters within the last 6 months was used to measure the size of a person’s core network. Among those who reported one or more core tie, a name interpreter instrument was used to ask respondents if their core ties lived (1) in their households, (2) in their neighbourhoods, (3) within the MMA, or (4) outside the MMA. Responses were recoded for this analysis to compare the chances of depressive symptoms in those adults who had (1) no core ties, (2) neighbourhood ties only, (3) non-neighbourhood ties only (i.e., ties within the household only or outside the neighbourhood only), or (4) mixed source ties (i.e., both neighbourhood and non-neighbourhood ties). These categorizations allowed for the comparison of individuals with neighbourhood core ties only to respondents in each of the other three categories.

#### Social cohesion

Social cohesion was assessed along three dimensions: (1) generalized trust, (2) trust in neighbours, and (3) perceived neighbourhood cohesion. Generalized trust in others was measured by asking respondents “Generally speaking, would you say that most people can be trusted or that you can’t be too careful in dealing with people?” Possible response options included: (1) most people can be trusted, (2) can’t be too careful, (3) depends, (4) most people cannot be trusted, and (5) don’t know. Responses were coded dichotomously so those who answered “most people can be trusted” could be compared to those with lower levels of trust. This is the most common used question to measure trust in the health literature, and has been shown in experimental studies to correlate with honesty [31].

To measure “trust in neighbours,” participants were given the statement: “Most people in your neighbourhood can be trusted.” Based on a five-point Likert scale, respondents replied from strongly disagree to strongly agree. These responses were centered on 0 as the neutral category and coded so that a higher score represented higher trust in neighbours.

Respondents reported the extent to which they agreed with the following four statements as an indicator of perceived neighbourhood cohesion: (1) people in your neighbourhood are willing to help each other, (2) most people in your neighbourhood know you, (3) you have trouble with your neighbours, and (4) your neighbourhood is clean. Responses were recorded on a five-point Likert scale from “strongly disagree” to “strongly agree.” Principal components analysis was used to construct a perceived neighbourhood cohesion scale. The four items loaded at 0.51, 0.42, 0.16, and −0.38, respectively, suggesting that the score primarily reflected participant’s sense of neighbourhood cohesion.

#### Socio-demographic characteristics

Socio-demographic and -economic variables included measures of gender, age, marital status, foreign-born status, primary household language, education, income, and employment status. Gender was measured dichotomously, with respondents have reported whether they were male or female. Age was measured with six age categories, including: (1) 25–34 years old, (2) 35–44 years old, (3) 45–54 years old, (4) 55–64 years old, (5) 65–74 years old, and (6) 75 years and older. Marital status categories were (1) currently married or in a common law relationship, (2) single, (3) separated, (4) divorced, and (5) widowed. Foreign-born status was measured by asking participants if they were born inside or outside Canada, and primary household language was measured by asking respondents if they most often spoke French, English, or other languages within their household. Education included four categories: (1) no high school certificate or diploma, (2) high school diploma or equivalent and trade certificate or diploma, (3) college certificate or diploma lower than a bachelor’s degree, (4) bachelor’s degree and higher. Income was divided into five categories: (1) less than $28 000, (2) $28 000 to 49 000, (3) $50 000 to $74 000, (4) $75 000 to $100 000, (5) over $100 000. To account for missing data on income for 20% of respondents, income data were imputed for these cases using an ordinal regression. Income data were imputed based on respondent education, employment status, age, and the median household income of the census tract in which the respondent resided.

Census-tract level measures of neighbourhood disadvantage were constructed using data from the 2006 Canada Census. The neighbourhood disadvantage score was composed of a standardized factor score of census-tract unemployment rates, percentage of immigrants, percentage of single mothers, and percentage of renters. Their proportional contributions to the disadvantage score were 0.69, 0.18, 0.11, and 0.03 respectively. Neighbourhood population density was based on the 2006 Canada Census census-tract level estimates of population size per square kilometer. Given that the sample of MMA neighbourhoods covered more urban and suburban environments, population density was used to help adjust for potential differences in neighbourhood physical environments that may not have been due to variations in disadvantage.

### Statistical Analyses

Multilevel logistic regression was used to examine the contextual level association of disadvantage with depressive symptoms while adjusting for individual compositional factors. First, the overall variance between neighbourhoods in having depressive symptomatology was estimated and reported as the intraclass correlation coefficient (ICC) using the full MoNNET-HA sample data. The ICC for a dichotomous outcome was calculated as:
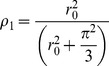
where ρ_I_ is the ICC value, r_0_^2^ is the intercept variance, and π is 3.14159 (Snijders and Bosker, 1999). A 95% plausible value range (PVR) for the ICC was also calculated to describe the range of variability between tracts in having depressive symptomatology. The ICCs of depressive symptoms were calculated for men and women separately. Second, to reduce potential confounding of the relationships between disadvantage, social capital and depressive symptoms by gender, separate multilevel logistic regressions were conducted for men and women. For each gender, a two-stage model-building process was used. The first models assessed whether neighbourhood disadvantage was associated with depressive symptoms after controlling for neighbourhood density and the individual socio-demographic and -economic compositional characteristics. Social capital variables were added in the second models to examine which components of social capital were associated with depressive symptoms in men and women. Likelihood ratio (LR) tests were used to compare the fit of models one and two. Changes in the ICC from the null models to models one and two were not evaluated due to the low sample size of either men or women within each tract in the stratified analyses.

If results from models one and two suggested potential mediation, mediation tests were conducted to identify which social capital variables potentially mediated the association between disadvantage and depressive symptoms. The three-step multilevel mediation process used by Krull and MacKinnon was employed [32]. First, using multilevel linear regression, neighbourhood disadvantage was regressed on each separate social capital component while adjusting for socio-demographic and -economic characteristics. The coefficients for these associations were retrieved, and labelled “a”. Second, using multilevel logistic regression, having or not having depressive symptoms was regressed on the separate social capital components. These estimates were labelled “b.” Third, the product of these coefficients, ab, were calculated to provide an estimate of the mediated effects for each of social capital variables. The separate ab estimates and their standard errors were used to calculate z-scores, Wald statistics, and 95% confidence intervals.

MoNNET-HA response rates were estimated at 38.7% using the second response rate definition from the American Association of Public Opinion Research. Using data from the 2006 Canada Census, chi-square analyses were conducted to assess the representativeness of the MoNNET-HA sample. Results showed that the MoNNETs-HA study over-represented women, older adults (by study design), and persons with incomes under $50,000, those who had lived in their current place of residence for over 5 years, and those with college and university education [29]. After excluding observations from participants who were missing information on any of the main study variables, the final sample size for this study was 2574 participants, or 912 men and 1662 women.

## Results

The overall prevalence of having depressive symptoms in the MoNNET-HA sample was 17.3%. The prevalence of depression in women was 19.5%; the prevalence was lower in men at 13.4%. The overall variability in having depressive symptomatology between MMA census tracts was estimated to have a neighbourhood ICC of 8.00% (95% PVR: 2.77, 12.78). Among men, the neighbourhood ICC was 10.5% (95% PVR: −6.45, 22.76); among women, the ICC was 8.00% (95% PVR: 0.95, 14.11). Descriptive information on the sample is provided in Table S1. Main results for the stratified regression analyses are found in Tables 1 and 2. Results of socio-demographic and –economic characteristics with depressive symptoms may be seen in Tables S2(men) and S3(women). Summary findings for men and women are below.

**Table 1:** Adjusted odds ratio and 95% confidence intervals from multilevel logistic regression analyses, MoNNET-HA Men, n = 912 (controlling for socio-demographic characteristics).

	Model 1a	Model 2a
**Neighbourhood-level variables**		
Neighbourhood disadvantage	0.96 (0.68–1.35)	0.84 (0.59–1.20)
Neighbourhood population density	1.01 (0.95–1.07)	1.01 (0.95–1.07)
**Individual-level variables**		
**Social capital dimensions**		
**Core tie diversity**		
No core ties	–	0.77 (0.36–1.68)
Neighbourhood and non neighbourhood ties	–	1.43 (0.65–3.13)
No neighbours as core tie	–	0.71 (0.29–1.76)
Neighbourhood ties only	–	1.00
**Generalized trust**		
Low trust	–	1.42 (0.87–2.31)
High trust	–	1.00
**Trust in neighbours**	–	0.80 (0.67–0.95)*
**Perceived neighbourhood cohesion factor**	–	0.64 (0.44–0.94)*
**Likelihood ratio test**	–	26.90***

* p<0.05,

** p<0.01,

*** p<0.001.

**Table 2:** Adjusted odds ratio and 95% confidence intervals from multilevel logistic regression analyses, MoNNET-HA Women, n = 1662 (controlling for socio-demographic characteristics).

	Model 1b	Model 2b
**Neighbourhood-level variables**		
Neighbourhood disadvantage	1.25 (1.01–1.55)*	1.18 (0.94–1.47)
Neighbourhood population density	0.98 (0.95–1.02)	0.98 (0.94–1.02)
**Social capital variables:**		
**Core tie diversity**		
No core ties	–	1.79(1.05–3.06)*
Neighbourhood and non-neighbourhood ties	–	2.28 (1.32–3.94)**
No core neighbourhood ties	–	1.34 (0.71–2.50)
Neighbourhood ties only	–	1.00
**Generalized trust**		
Low trust	–	1.54 (1.13–2.08)**
High trust		1.00
**Trust in neighbours**	–	0.88 (0.79–0.98)*
**Perceived neighbourhood cohesion factor**	–	0.68 (0.54–0.85)**
**Likelihood ratio test**	–	44.40***

* p<0.05,

** p<0.01,

*** p<0.001.

### Men

Table 1 provides the adjusted odds ratios and 95% confidence intervals of the likelihood of depressive symptoms in men due to neighbourhood disadvantage and social capital.

#### Model 1a: Neighbourhood disadvantage and socio-demographic and -economic factors

Neighbourhood disadvantage was not associated with reports of depressive symptoms in men. Certain individual socio-demographic and -economic characteristics were associated with depressive symptoms (Please refer to Table S2). For example, men over 75 were 78% less likely (OR = 0.22, 95% CI = 0.07–0.70) to have depressive symptoms than men aged 25–34 years old. Men who were single (OR = 2.92; 95% CI = 1.66–5.15), divorced or separated (OR = 2.14; 95% CI = 1.05–4.37), or widowed (OR = 7.75; 95% CI = 3.24–18.55) were more likely to report depressive symptoms than men who were married or in a common-law relationship.

#### Model 2a: Social cohesion and social capital

Men with more positive perceptions of neighbourhood cohesion were less likely to report depressive symptoms than men who viewed their neighbourhoods less favourably (OR = 0.64; 95% CI = 0.44–0.94). In addition, men with higher trust in neighbours were less likely to report depressive symptoms than those with lower trust (OR = 0.80; 95% CI = 0.67–0.95). The LR test indicated that Model 2a provided a better fit with depressive symptoms in men than those in Model 1a (LR test = 26.90, p<0.001).

### Women

Table 2 provides the adjusted odds ratios and 95% confidence intervals of the likelihood of depressive symptoms in women due to neighbourhood disadvantage and social capital.

#### Model 1b: Neighbourhood disadvantage and socio-demographic and -economic factors

Neighbourhood disadvantage was positively associated with the odds of having depressive symptoms in women (OR = 1.25; 95% CI = 1.01–1.55). In addition, women aged 65–74 were 50% less likely (OR = 0.50; 95% CI = 0.29–0.85) to have depressive symptoms than those aged 25–34 years old. Table S3 provides the odds ratios and 95% CIs on the association between depression and socio-demographic and -economic variables. Marital status was associated with the likelihood of having depressive symptoms in women. Women who were divorced or separated were more likely to report depressive symptoms than women who were married or in a common-law relationship (OR = 2.18; 95% CI = 1.48–3.20). Those who were widowed were also more likely to report depressive symptoms (OR = 1.60; 95% CI = 0.98–2.63). In general, higher income decreased women’s odds of being depressed with those making over $28,000 less likely to have depressive symptoms compared to those making under $28,000.

#### Model 2b: Social cohesion and social capital

Having positive perceptions of neighbourhood cohesion decreased the likelihood of having depressive symptoms in women (OR = 0.68; 95% CI = 0.54–0.85). Women who had higher trust in neighbours were less likely to report depressive symptoms than women with low trust in neighbours (OR = 0.88; 95% CI = 0.79–0.98). Women with low generalized trust (OR = 1.54; 95% CI = 1.13–2.08) were more likely to report depressive symptoms than those with high generalized trust. Women with both neighbourhood and non-neighbourhood ties were more likely (OR = 2.28; 95% CI = 1.32–3.94) to have depressive symptoms compared to those with ties only inside the neighbourhood. Women with no reported core ties were more likely to experience depressive symptoms than those with neighbourhood core ties (OR = 1.79; 95% CI = 1.05–3.06). The LR test indicated that variables in model 2b provided a better fit of depressive symptoms in women than variables in model 1b (LR test = 44.40, p<0.001).

### Mediation Analyses

Results from the mediation analyses may be seen in Table 3. Given that neighbourhood disadvantage was not shown associated with depressive symptoms in men, no mediation tests were conducted with the men’s data. Among women, perceived neighbourhood cohesion was the only social capital dimension among the four shown to mediate the association between neighbourhood disadvantage and depressive symptoms (ab = 0.02; 95% CI = 0.003–0.04, p<0.05).

**Table 3:** Mediation analyses: social capital variables as mediators of neighbourhood disadvantage and depressive symptoms in women, n = 1662.

Social Capital Components	a	b	aSE	bSE	ab	SE (ab)	z	Wald statistic	p	lower95% CI	upper95% CI
**Generalized Trust**	0.06	0.43	0.08	0.16	0.03	0.04	0.72	0.52	0.47	−0.04	0.10
**Trust in Neighbours**	−0.08	−0.13	0.04	0.06	0.01	0.01	1.47	2.16	0.14	−0.00	0.02
**Core Tie Diversity**(vs. neighbourhood ties)											
No neighbourhood ties	0.06	0.58	0.08	0.27	0.03	0.05	0.71	0.50	0.48	−0.06	0.13
Ties inside and outside the neighbourhood	0.01	0.82	0.09	0.28	0.00	0.07	0.06	0.00	0.96	−0.14	0.15
No core ties	−0.16	0.29	0.12	0.32	−0.05	0.06	−0.75	0.56	0.45	−0.17	0.07
**Perceived neighbourhood cohesion factor**	−0.06	−0.39	0.02	0.12	0.02	0.01	2.20	4.86	0.03*	0.00	0.04

* p<0.05.

## Discussion

The current study investigated whether the potential social pathways linking neighbourhood disadvantage to depressive symptoms were similar in men and women. The study also assessed whether aspects of neighbourhood social cohesion and social capital were associated with depressive symptoms in women and men in the same way. Among women, findings showed that (1) higher levels of neighbourhood disadvantage increased the odds of reporting depressive symptoms, (2) aspects of neighbourhood social cohesion and social capital were associated with depressive symptoms, and (3) perceived neighbourhood cohesion fully mediated the association of disadvantage with depressive symptoms. Among men, results demonstrated that (1) disadvantage was not associated with depressive symptoms in men and (2) similar to women, aspects of social cohesion and social capital were associated with depressive symptoms.

Our findings suggest, first of all, that there may be important differences in the way that neighbourhood disadvantage relates to depressive symptomatology in men and women. In our sample, neighbourhood disadvantage was associated with depressive symptoms in women but not in men. To our knowledge, only one previous study has directly investigated gender differences in neighbourhood disadvantage and depressive symptoms [14]. In that study, neighbourhood disadvantage (measured with indicators of residential instability, material deprivation, dependency, and ethnic diversity) was associated with depression, but no differences were found between men and women [14]. One possible explanation as to why associations were found in the current study but not in the one conducted previously may be due to sample and measurement differences. Matheson et al.’s study examined associations between disadvantage and depression in a range of cities and not exclusively one metropolitan area and used slightly different indicators of neighbourhood disadvantage [14]. It is possible that associations may be dependent on (a) the urban environment examined and (b) the specific indicators of neighbourhood disadvantage measured. Due to the limited number studies of gender, neighbourhood disadvantage, and depression, it may be helpful to turn to studies that have examined whether the association between neighbourhood characteristics and other health outcomes varies in men and women. For example, Karriker-Jaffe (2009) reported that neighbourhood socioeconomic disadvantage was predictive of aggression in adolescent girls but not boys [33]. Stafford and colleagues reported that the magnitude of the association between neighbourhood characteristics, such as the built environment and economic deprivation, and poor self-rated health was greater in women than men [7]. Although research in this specific area continues to emerge, our study supports the idea that untoward neighbourhood conditions may have a stronger impact on the mental health of women.

In assessing the social pathways by which disadvantage may relate to depressive symptoms in women, our study found perceived neighbourhood cohesion to be the only factor that might operate as an intermediary mechanism. As such, our findings suggest that neighbourhood disadvantage may relate to depressive symptoms in women via cognitive, psychosocial pathways. In slight contrast to our findings, however, Haines et al. (2011) found network components of social capital to mediate the association between neighbourhood disadvantage and depressive symptoms in a combined sample of men and women in a mid-sized city in the southern United States [9]. Differences in whether psychosocial or network resource mechanisms link disadvantage to depressive symptoms may be contextually or situationally specific. In contexts or situations (e.g., disasters) of greater resource scarcity, resource mechanisms may play a greater role than psychosocial ones. Future research might consider the importance of the broader macro-political environment in assessing the relative influence of specific pathways linking neighbourhood disadvantage to depressive symptoms.

Despite differences between men and women in the importance of neighbourhood disadvantage, men and women did share certain patterns at the interpersonal level in how social cohesion and social capital was associated with depressive symptoms. As shown in other studies, lower trust in neighbours and lower perceived neighbourhood cohesion increased the odds of depressive symptoms in both men and women [18], [21], [24]. There were also differences between women and men at the interpersonal level. Low generalized trust was shown to increase the odds of depressive symptoms in women only.

Furthermore, diversity in the geographical source of one’s core ties was shown to be important only in women. First, women with neighbourhood core ties were less likely to experience depressive symptoms than women who reported no core ties or ties both inside and outside the neighbourhood. The former finding is consistent with research on social isolation and depression that has shown that individuals with no close ties tend to report higher rates of depression than those who report more close ties [20]-[24]. The latter finding is less understood but suggests the potential importance for women of more spatially proximate ties. To our knowledge, no other studies have investigated gender differences within this component of social capital. However, recent literature supports the mental health benefits of having positive relationships within the neighbourhood [16], [26]. Thus, it may be that having all core ties within close proximity may be particularly beneficial for women’s mental health. Future research may wish to further investigate why women who have more spatially diverse ties are more likely to have depressive symptoms than those with ties solely within the neighbourhood.

### Limitations

The current study had some unavoidable limitations. First, depressive symptoms were not clinician-rated. Although the CES-D-10 is a valid and reliable screening tool for measuring depressive symptoms, it is not able to make clinical diagnoses of depression [34]. As a result, conclusions in this study are limited to depressive symptoms, but not major depressive disorder. Second, there may be a discrepancy between the study’s objective definition of neighbourhoods in terms of census tracts and the participant’s subjective perceptions of their neighbourhood boundaries. Whereas neighbourhood-level disadvantage was measured from census tract data, the individual-level measure of neighbourhood perception was based on respondent’s own mental frames of where their neighbourhood boundaries lie. It may be that respondent’s perceptions differed from the strictly defined census tract boundaries. Third, the perceived cohesion items had low reliability (α = 0.32) in scalar form. As a result, principal components analysis was used to construct a perceived cohesion variable. Sensitivity analyses were conducted comparing findings using the original perceived neighbourhoods component score with the cohesion scale and again with the cohesion items separately. These analyses yielded similar results as presented here in the original analysis. Fourth, Krull and Mackinnon’s multilevel mediation models are based on continuous outcomes. The application of these methods to logistic models is less understood. However, Kenny considers the problem of extending these methods to binary outcomes as likely small [35]. Lastly, due to the study’s cross-sectional design, causality cannot be inferred. Cross-sectional mediation tests are useful in evaluating potential causal pathways linking neighbourhood environments to depressive symptomatology. Although individual perceptions of the neighbourhood environment or depression are not likely to influence contextual-level neighbourhood disadvantage, depression may have an influence on a person’s perception of neighbourhood cohesion.

### Conclusion

To our knowledge, this is the first study to investigate gender differences in the association between neighbourhood disadvantage and depression and whether neighbourhood social cohesion and social capital mediated such links in women. Neighbourhood disadvantage was associated with depressive symptoms in women only, and these associations were fully mediated by their perceptions of the neighbourhood environment. Findings from this study suggest that gender differences should be considered in studies of neighbourhood disadvantage and depression. Moreover, health promotion or policy interventions targeting neighbourhood disadvantage may consider the inclusion of gender-specific strategies addressing the mental health of women and men.

## Supporting Information

Table S1 **Characteristics of Montreal Neighbourhood Networks and Healthy Aging study (MoNNET-HA), social capital and depressive symptoms sample, n = 2574.**
(DOCX)

Table S2 **Adjusted odds ratio and 95% confidence intervals of socio-demographic characteristics from multilevel logistic regression analyses, MoNNET-HA Men, n = 912 (Model 1a and Model 2a).**
(DOCX)

Table S3 **Adjusted odds ratio and 95% confidence intervals of socio-demographic characteristics from multilevel logistic regression analyses, MoNNET-HA Women, n = 1662 (Model 1b and Model 2b).**
(DOCX)
