# Supplementary material for: Gender Differences in the Social Pathways Linking Neighborhood Disadvantage to Depressive Symptoms in Adults
Source: PLoS One. 2013 Oct 17;8(10):e76554. doi: 10.1371/journal.pone.0076554 (PMC3798396; doi:10.1371/journal.pone.0076554)
Supplement: Table S1 — Characteristics of Montreal Neighbourhood Networks and Healthy Aging study (MoNNET-HA), social capital and depressive symptoms sample, n = 2574. (DOCX) [file pone.0076554.s001.docx]

Table S1. Characteristics of Montreal Neighborhood Networks and Healthy Aging study (MoNNET-HA) by gender, social capital and depressive symptoms' sample, n=2574.

| **Variables** | **Percentage** | |
| --- | --- | --- |
|  | **Men (N= 912)** | **Women (N = 1662)** |
| **Depressive symptoms** | 13.4 | 19.5 |
| **Age** |  |  |
| 25-34 | 13.5 | 15.7 |
| 35-44 | 19.6 | 17.0 |
| 45-54 | 21.7 | 19.7 |
| 55-64 | 16.1 | 16.1 |
| 65-74 | 20.1 | 21.1 |
| 75+ | 9.0 | 10.4 |
| **Marital Status** |  |  |
| Married | 57.6 | 53.1 |
| Single | 24.0 | 18.7 |
| Divorced/Separated | 12.4 | 15.9 |
| Widowed | 6.0 | 12.3 |
| **Education** |  |  |
| No high school | 8.3 | 13.4 |
| High School/Trade | 28.2 | 30.0 |
| College | 19.6 | 21.3 |
| University degree or higher | 43.9 | 35.3 |
| **Income** |  |  |
| Less than $28,000 | 15.6 | 22.4 |
| $28,000-49,000 | 27.9 | 28.5 |
| $50,000-74,000 | 27.2 | 26.8 |
| $75,000-100,000 | 14.5 | 12.3 |
| $100,000 and over | 14.9 | 10.0 |
| **Foreign born status** | 20.8 | 17.2 |
| **Household language** |  |  |
| French | 76.9 | 78.6 |
| English | 14.6 | 13.5 |
| Foreign language | 8.6 | 7.9 |
| **Unemployed** | 38.9 | 48.1 |
| **Social Capital Dimensions** |  |  |
| **Core tie diversity** |  |  |
| Neighborhood ties only | 9.4 | 9.1 |
| Non-neighborhood ties only | 45.3 | 44.5 |
| Neighborhood and non-neighborhood ties | 31.5 | 33.7 |
| No core ties | 13.8 | 12.8 |
| **Generalized trust** |  |  |
| High trust | 48.9 | 39.5 |
| Low trust | 51.1 | 60.5 |
