# Supplementary material for: Gender Differences in the Social Pathways Linking Neighborhood Disadvantage to Depressive Symptoms in Adults
Source: PLoS One. 2013 Oct 17;8(10):e76554. doi: 10.1371/journal.pone.0076554 (PMC3798396; doi:10.1371/journal.pone.0076554)
Supplement: Table S2 — Adjusted odds ratio and 95% confidence intervals of socio-demographic characteristics from multilevel logistic regression analyses, MoNNET-HA Men, n = 912 (Model 1a and Model 2a). (DOCX) [file pone.0076554.s002.docx]

Table S2: Adjusted odds ratio and 95% confidence intervals of socio-demographic characteristics from multilevel logistic regression analyses, MoNNET-HA Men, n=912 (Model 1a and Model 2a).

| **Socio-demographic and -economic variables** | **Model 1a** | **Model 2a** |
| --- | --- | --- |
| **Age category** |  |  |
| 75+ | 0.22 (0.07-0.70)** | 0.32 (0.10-1.03) |
| 65-74 | 0.59 (0.25-1.39) | 0.70 (0.30-1.64) |
| 55-64 | 0.46 (0.19-1.08) | 0.52 (0.22-1.24) |
| 45-54 | 0.94 (0.46-1.95) | 1.02 (0.49-2.13) |
| 35-44 | 1.02 (0.49-2.10) | 1.13 (0.55-2.34) |
| 25-34 | 1.00 | 1.00 |
| **Marital status** |  |  |
| Widowed | 7.75 (3.24-18.55)*** | 8.34 (3.40-20.46)*** |
| Single | 2.92 (1.66-5.15)*** | 2.93 (1.66-5.19)*** |
| Divorced/Separated | 2.14 (1.05-4.37)* | 1.96 (0.95-4.05) |
| Married | 1.00 | 1.00 |
| **Education** |  |  |
| No degree | 0.78 (0.42-1.43) | 0.74 (0.30-1.83) |
| High School/Trade | 0.80 (0.46-1.41) | 0.77 (0.43-1.38) |
| College | 0.78 (0.33-1.85) | 0.77 (0.41-1.44) |
| University degree | 1.00 | 1.00 |
| **Income** |  |  |
| $100,000 and over | 0.51 (0.19-1.36) | 0.55 (0.20-1.52) |
| $75,000-100,000 | 0.61 (0.24-1.56) | 0.62 (0.24-1.59) |
| $50,000-74,000 | 0.66 (0.32-1.36) | 0.66 (0.31-1.38) |
| $28,000-49,000 | 0.86 (0.46-1.60) | 0.87 (0.46-1.65) |
| Less than $28,000 | 1.00 | 1.00 |
| **Foreign born status** |  |  |
| Born outside of Canada | 1.29 (0.71-2.35) | 1.31 (0.71-2.41) |
| Born in Canada | 1.00 | 1.00 |
| **Household language** |  |  |
| English | 1.63 (0.89-3.00) | 1.82 (0.97-3.40) |
| Foreign language | 1.23 (0.53-2.87) | 1.22 (0.52-2.83) |
| French | 1.00 | 1.00 |
| **Employment status** |  |  |
| Not currently employed | 1.65 (0.92-2.95) | 1.60 (0.89-2.88) |
| Employed | 1.00 | 1.00 |

*p<0.05, **p<0.01, ***p<0.001
