# Supplementary material for: Gender Differences in the Social Pathways Linking Neighborhood Disadvantage to Depressive Symptoms in Adults
Source: PLoS One. 2013 Oct 17;8(10):e76554. doi: 10.1371/journal.pone.0076554 (PMC3798396; doi:10.1371/journal.pone.0076554)
Supplement: Table S3 — Adjusted odds ratio and 95% confidence intervals of socio-demographic characteristics from multilevel logistic regression analyses, MoNNET-HA Women, n = 1662 (Model 1b and Model 2b). (DOCX) [file pone.0076554.s003.docx]

Table S3: Adjusted odds ratio and 95% confidence intervals of socio-demographic characteristics from multilevel logistic regression analyses, MoNNET-HA Women, n=1662 (Model 1b and Model 2b)

| **Socio-demographic and -economic variables** | **Model 1b** | **Model 2b** |
| --- | --- | --- |
| **Age category** |  |  |
| 75+ | 0.52 (0.28-1.00) | 0.63 (0.33-1.23) |
| 65-74 | 0.50 (0.29-0.85)* | 0.58 (0.34-1.01) |
| 55-64 | 0.79 (0.48-1.28) | 0.89 (0.54-1.47) |
| 45-54 | 0.97 (0.62-1.52) | 0.95 (0.60-1.49) |
| 35-44 | 0.78 (0.49-1.24) | 0.82 (0.51-1.32) |
| 25-34 | 1.00 | 1.00 |
| **Marital status** |  |  |
| Single | 1.41 (0.97-2.05) | 1.39 (0.95-2.04) |
| Divorced/Separated | 2.18 (1.48-3.20)*** | 2.23 (1.50-3.32)*** |
| Widowed | 1.60 (0.98-2.63) | 1.69 (1.02-2.80)* |
| Married | 1.00 | 1.00 |
| **Education** |  |  |
| No degree | 1.06 (0.66-1.69) | 1.01 (0.62-1.64) |
| High School/Trade | 0.96 (0.66-1.39) | 0.87 (0.59-1.28) |
| College | 0.78 (0.53-1.15) | 0.73 (0.49-1.08) |
| University degree | 1.00 | 1.00 |
| **Income** |  |  |
| $100,000 and over | 0.48 (0.25-0.92)* | 0.56 (0.28-1.09) |
| $75,000-100,000 | 0.58 (0.32-1.04) | 0.65 (0.35-1.19) |
| $50,000-74,000 | 0.57 (0.37-0.89)* | 0.64 (0.41-1.01) |
| $28,000-49,000 | 0.66 (0.46-0.95)* | 0.72 (0.50-1.05) |
| Less than $28,000 | 1.00 | 1.00 |
| **Foreign born status** |  |  |
| Born outside of Canada | 1.26 (0.84-1.90) | 1.37 (0.90-2.08) |
| Born in Canada | 1.00 | 1.00 |
| **Household language** |  |  |
| English | 0.83 (0.55-1.26) | 0.89 (0.59-1.36) |
| Foreign language | 1.02 (0.59-1.79) | 0.92 (0.52-1.63) |
| French | 1.00 | 1.00 |
| **Employment status** |  |  |
| Not currently employed | 1.18 (0.83-1.67) | 1.18 (0.82-1.68) |
| Employed | 1.00 | 1.00 |

*p<0.05, **p<0.01, ***p<0.001
